# Supplementary material for: Hyperspectral Imaging and Chemometrics for Authentication of Extra Virgin Olive Oil: A Comparative Approach with FTIR, UV-VIS, Raman, and GC-MS
Source: Foods. 2023 Jan 17;12(3):429. doi: 10.3390/foods12030429 (PMC9914562; doi:10.3390/foods12030429)
Supplement: Supplementary file 1 [file foods-12-00429-s001.zip › foods-2119813-supplementary.pdf]

SUPPLEMENTARY MATERIALS: Hyperspectral imaging and chemometrics for authentication of extra virgin olive oil: A comparative approach with FTIR, UV-VIS, Raman, and GC-MS

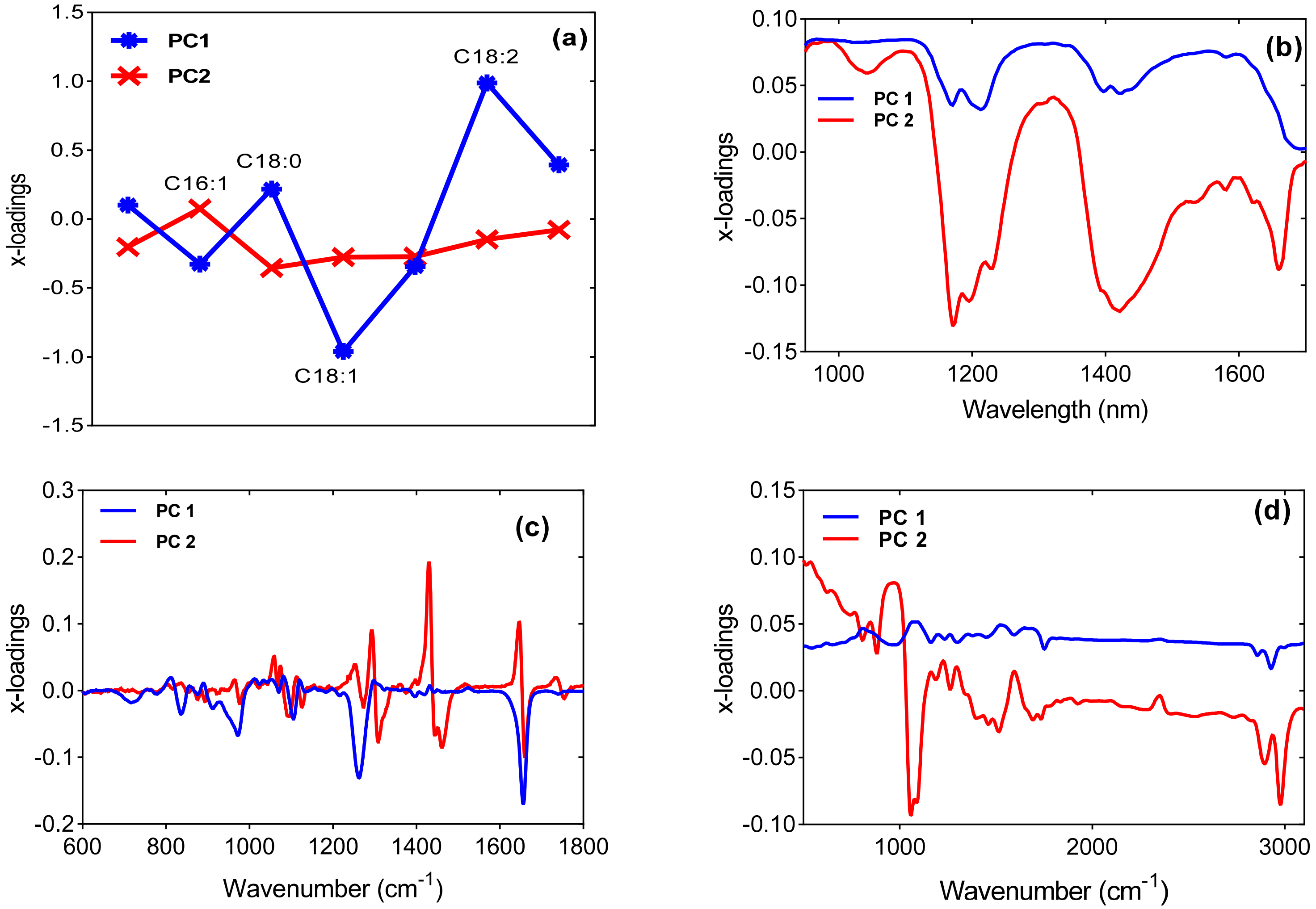

Figure S1. X-loadings showing variables that contribute to most of the variation among the principal components for all the techniques used in the study: **(a)** GC-MS, **(b)** Hyperspectral Imaging, **(c)** Raman, **(d)** FTIR

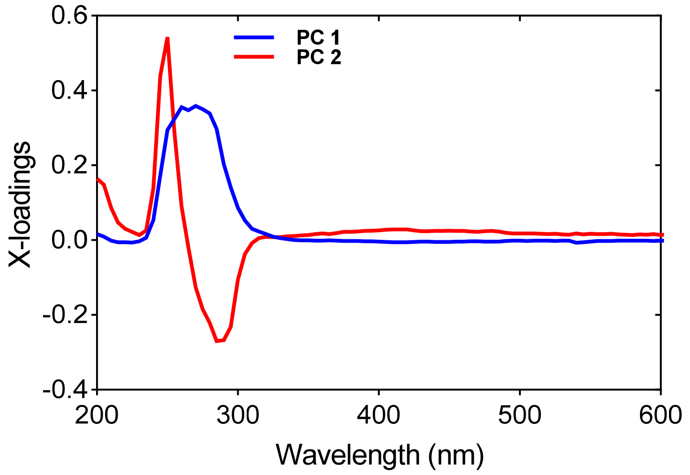

Figure S2. UV-Vis X-loadings for principal components 1 and 2

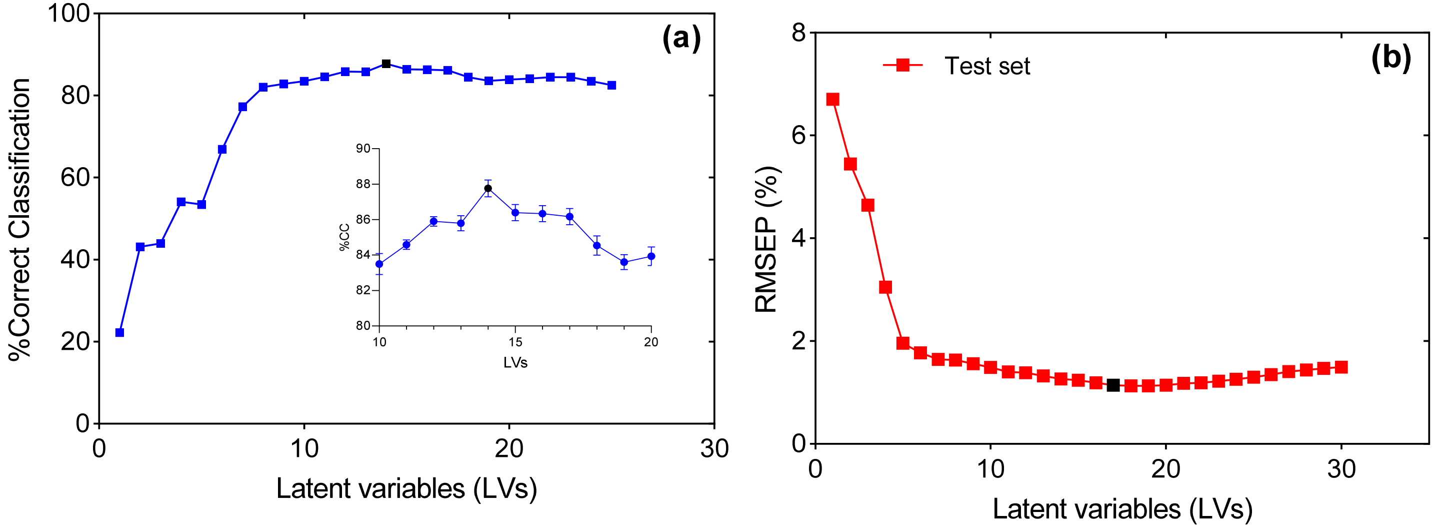

Figure S3. **(a)** Selection of optimum number of latent variables for correct classification (%CC ± S.E) with Raman and PLS-DA test models (**classification as pure EVOO, adulterant (type of edible oil) or as per category of adulterated EVOO)**. The minimum number of components selected for each model is highlighted in black; **(b)** Selection of optimum number of LVs based on RMSE± S.E for overall prediction (PLS regression) model from hyperspectral imaging technique. The optimum number of LVs is highlighted in black.

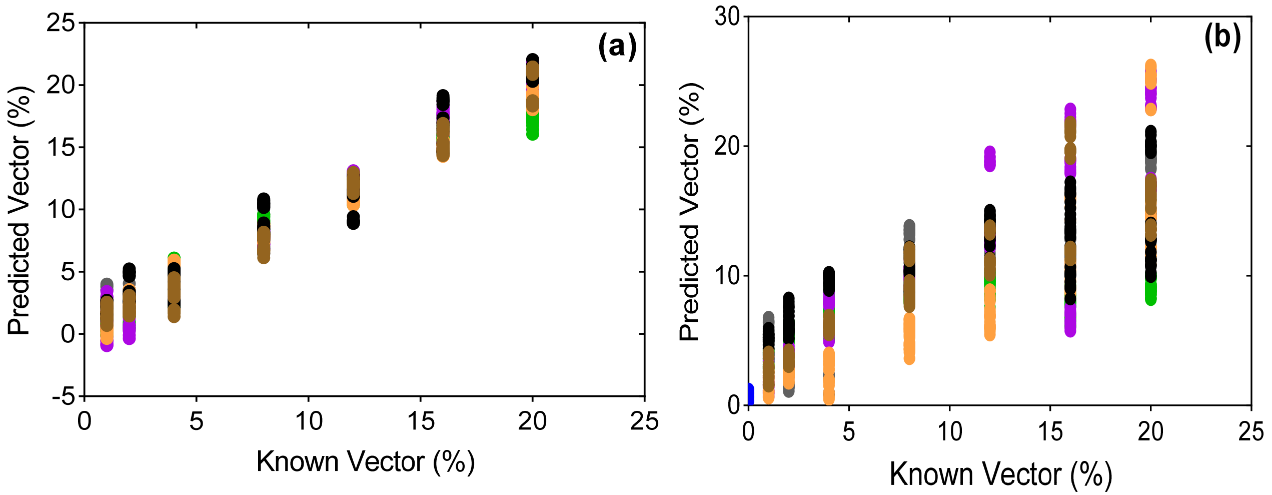

Figure S4. Variability in overall PLS prediction models: **(a)** HSI; **(b)** UV-Vis. • EVOO; • safflower oil; • corn oil; • soybean oil; • canola oil; • sunflower oil; • sesame oil
